# A Lesion-Aware Patch Sampling Approach with EfficientNet3D-UNet for Robust Multiple Sclerosis Lesion Segmentation

**DOI:** 10.3390/jimaging11100361

**Published:** 2025-10-13

**Authors:** Hind Almaaz, Samia Dardouri

**Affiliations:** 1 College of Computing and Information Technology, Shaqra University, Shaqra 11911, Saudi Arabia; s446500716@std.su.edu.sa; 2 InnoV’COM Laboratory-Sup’Com, University of Carthage, Ariana 2083, Tunisia

**Keywords:** multiple sclerosis, UNet3D, EfficientNet3D, lesion segmentation, MRI

## Abstract

Accurate segmentation of multiple sclerosis (MS) lesions from 3D MRI scans is essential for diagnosis, disease monitoring, and treatment planning. However, this task remains challenging due to the sparsity, heterogeneity, and subtle appearance of lesions, as well as the difficulty in obtaining high-quality annotations. In this study, we propose Efficient-Net3D-UNet, a deep learning framework that combines compound-scaled MBConv3D blocks with a lesion-aware patch sampling strategy to improve volumetric segmentation performance across multi-modal MRI sequences (FLAIR, T1, and T2). The model was evaluated against a conventional 3D U-Net baseline using standard metrics including Dice similarity coefficient, precision, recall, accuracy, and specificity. On a held-out test set, EfficientNet3D-UNet achieved a Dice score of 48.39%, precision of 49.76%, and recall of 55.41%, outperforming the baseline 3D U-Net, which obtained a Dice score of 31.28%, precision of 32.48%, and recall of 43.04%. Both models reached an overall accuracy of 99.14%. Notably, EfficientNet3D-UNet also demonstrated faster convergence and reduced overfitting during training. These results highlight the potential of EfficientNet3D-UNet as a robust and computationally efficient solution for automated MS lesion segmentation, offering promising applicability in real-world clinical settings.

## 1. Introduction

Multiple sclerosis (MS) is a chronic inflammatory demyelinating disease of the central nervous system characterized by scattered lesions in the brain and spinal cord, commonly detected using magnetic resonance imaging (MRI) [1]. The accurate segmentation of MS lesions is essential for diagnosis, disease progression monitoring, and therapeutic planning. Clinical MRI protocols typically include FLAIR (Fluid-Attenuated Inversion Recovery), and T1-weighted and T2-weighted sequences, each highlighting different lesion characteristics. However, manual delineation of lesions remains time-consuming and suffers from inter-rater variability, particularly when detecting small or subtle lesions [2].

Recent advances in deep learning, particularly convolutional neural networks (CNNs), have shown significant potential in automating MS lesion segmentation. The 3D U-Net architecture has emerged as a widely adopted baseline for volumetric neuroimaging tasks due to its ability to leverage spatial context. Nevertheless, its high computational cost and large parameter count limit its real-world deployment, especially in low-resource clinical settings [3]. Additionally, data imbalance where lesion voxels are vastly outnumbered by background tissue remains a persistent challenge in achieving high recall and precision [4]. To overcome these limitations, modern research has explored lightweight and scalable architectures such as EfficientNet, originally introduced for 2D image classification through compound scaling of depth, width, and resolution. While 2D EfficientNet backbones have been adapted for brain tumor segmentation and retinal imaging [5], their extension into full 3D volumetric networks for MS lesion detection is still underexplored. Moreover, most studies utilize only FLAIR inputs, ignoring the synergistic information offered by multi-modal MRI [6].

In this paper, we introduce a novel EfficientNet3D-based U-Net architecture tailored for multi-modal MS lesion segmentation using T1, T2, and FLAIR MRI volumes. Our approach integrates MBConv3D blocks with depthwise separable convolutions and trilinear upsampling, inspired by EfficientNetB4’s compound scaling principles. Unlike traditional UNet3D models, our architecture achieves competitive accuracy with fewer parameters and improved inference efficiency. We also implement a lesion-balanced patch extraction strategy and post-processing via small blob removal, designed to enhance recall and reduce false positives, especially important in detecting clinically relevant small lesions [6].

The primary contributions of this work are as follows:

- We introduce a novel and efficient 3D deep learning architecture based on EfficientNet3D, specifically designed for segmenting multiple sclerosis (MS) lesions using full 3D multi-modal MRI data, marking one of the first applications of this architecture in this domain.
- A lesion-aware patch sampling strategy is integrated with advanced data augmentation and a post-processing step involving small-blob suppression to suppress small false-positive predictions, thereby improving segmentation robustness.
- Comprehensive experiments on a clinically curated MS dataset demonstrate that our proposed model achieves consistently higher or competitive performance in Dice score, precision, and recall compared to the standard 3D U-Net baseline.

Together, these innovations present a scalable and accurate framework for MS lesion segmentation, supporting broader deployment of efficient 3D neural networks in clinical neuroimaging pipelines and future research.

## 2. Related Work

Recent developments in deep learning have yielded promising outcomes in automatic segmentation of multiple sclerosis (MS) lesions and brain tumors, particularly through the adoption of convolutional architectures fine-tuned for medical imaging. Nevertheless, challenges such as data heterogeneity, limited generalizability, and real-world deployment persist.

Wahlig et al. [3] explored transfer learning by fine-tuning 3D U-Nets on MS-specific lesion data. Their fine-tuned models outperformed de novo counterparts, achieving lesion-wise sensitivity of 0.75 and PPV of 0.75 on the ISBI 2015 dataset. While cross-validated Dice scores peaked at 0.66, results on external datasets such as ISBI without specific training showed a drop (e.g., Dice 0.62, sensitivity 0.56, PPV 0.61), revealing susceptibility to domain shifts. The reliance on small, non-diverse datasets also raises concerns regarding generalizability and overfitting. From a clinical deployment perspective, these limitations imply the need for larger, more representative training cohorts and cross-site validation.

Ashtari et al. [4] proposed Pre-U-Net, a variant of the traditional U-Net architecture with pre-activation residual blocks and deep supervision, evaluated on the MSSEG-2 dataset for longitudinal MS lesion segmentation. Their model achieved a Dice score of 45.6%, sensitivity of 54.5%, precision of 53.8%, and F1-score of 51.9%, outperforming U-Net and Res-U-Net baselines in detecting new lesions. However, performance deteriorated for extremely subtle or borderline lesions, particularly those missed by all raters, indicating a limitation in handling clinically ambiguous cases. This model’s sensitivity may fall short of clinical needs in real-world longitudinal monitoring, where early lesion evolution is subtle and detection critical.

Krishnan et al. [5] proposed a multi-arm 3D U-Net with dense input concatenation and skip connectivity for T2 lesion segmentation in MS clinical trials. The model achieved a Dice similarity coefficient of 0.66 and a lesion-wise sensitivity of 0.84, demonstrating robust performance across both internal and external datasets. However, the model’s reliance on large, multicenter clinical trial data may limit its adaptability to small-scale or heterogeneous real-world datasets, where data augmentation and domain adaptation be-come essential.

McKinley et al. [7] compared a standard 3D U-Net and DeepSCAN for joint lesion and brain structure segmentation. Their study reported a Dice score of 0.57 on the MSSEG dataset, which dropped to 0.49 when evaluated on an external cohort. This highlights the prevalent issue of domain shift, where models trained on single-center data underperform when exposed to external distributions. Such results stress the need for architecture and training strategies that promote cross-domain generalizability.

Davarani et al. [8] developed an enhanced segmentation approach utilizing DeepLabV3Plus with a Squeeze-and-Excitation (SE) module and an EfficientNetB0 back-bone. Focusing on FLAIR images, their model demonstrated superior performance, achieving a Dice score of 76.24, precision of 88.89%, and recall of 73.52% on a dataset comprising 1500 labeled slices from 100 MS patients. This study underscores the efficacy of integrating attention mechanisms and advanced backbone architectures in improving segmentation accuracy. However, the use of only 2D axial slices inherently limits the model’s ability to capture 3D lesion morphology and continuity, which are crucial in MS diagnosis. Moreover, the dataset used, though sizable in slice count, originated from a single cohort and imaging protocol, raising concerns about generalizability across scanners and institutions. In real-world deployment, the model’s performance may degrade on unseen modalities or in cases involving subtle lesion evolution over time, highlighting the necessity of volume-aware training and domain adaptation strategies.

Raab et al. [9] proposed a multi-modal convolutional neural network (CNN) architecture with distinct encoder branches for each MRI modality (T1, T2, and FLAIR), enabling modality-specific feature extraction while avoiding weight sharing. The model was optimized for low-resource environments and achieved a Dice score of 0.64, a precision of 0.67, and a sensitivity of 0.61 on the ISBI 2015 dataset. The inference time per volume was under 90 s on CPU-only hardware, illustrating its suitability for deployment in non-GPU clinical settings. However, the model relied on 2D axial slices, which impaired its ability to model inter-slice spatial continuity and led to missed detections of small and fragmented lesions, particularly in deep white matter regions.

Preetha et al. [10] introduced a brain tumor segmentation framework based on Effi-cientNetB4 with multiscale attention. On the Figshare dataset, the model achieved a Dice coefficient of 0.9339, IoU of 0.8795, precision of 0.9657, recall of 0.9103, specificity of 0.9996, and overall accuracy of 99.79%. These results demonstrate superior segmentation fidelity, particularly in binary tumor localization. However, the study was constrained to binary segmentation without validation on multiclass or lesion-specific MS tasks, limiting its translational applicability. Additionally, while the model’s performance was reported using key metrics such as precision, recall, and Dice score, it lacked an assessment of model interpretability an essential factor for real-time radiological integration.

These studies illustrate the significant advancements achieved in medical image segmentation through deep learning, particularly in the context of multiple sclerosis (MS) lesion detection, as summarized in Table 1. However, persistent issues such as domain generalization, sensitivity to small or subtle lesions, and the lack of scalable, resource-efficient solutions continue to hinder clinical adoption. These limitations underscore the need for segmentation frameworks that balance architectural efficiency, lesion-level sensitivity, and robustness across multi-modal MRI inputs and diverse clinical scenarios, an objective central to the present study. Recent advances in multiple sclerosis lesion segmentation have predominantly utilized deep learning frameworks based on U-Net architectures, enhanced with 3D modeling, attention mechanisms, residual connections, and recurrent units to improve accuracy and robust-ness. For instance, Zhang et al. in [11] proposed EfficientNet3D-UNet with lesion-aware patch sampling to focus on lesion regions, while Sharma [12] introduced a 3D recurrent residual attention U-Net to better capture spatial context. Other work, such as Abdelrah-man & El-Sayed [13] and Rondinella et al. [14], leveraged gated attention and adaptive feature weighting to boost segmentation performance. Approaches like Sarica & Tuncer [15] and Ashtari et al. [16] focused on dense residual connections and pre-activation schemes for enhanced feature extraction. To address variability and data scarcity, Bai et al. [17] applied federated learning with noise-resilient training, and Cetin et al. [18] used data augmentation strategies. Transfer learning and wavelet-based preprocessing have also been explored [19,20] to improve model generalization and lesion delineation. Additionally, large-scale evaluations and competitions [21] alongside multicentric validation studies [22] have contributed to advancing clinically viable MS lesion segmentation methods.

## 3. Materials and Methods

### 3.1. Dataset Description

This study utilized the publicly available Brain MRI Dataset of multiple sclerosis with Consensus Manual Lesion Segmentation and Patient Meta Information, accessible via the Mendeley Data repository. The dataset comprises multi-sequence MRI scans and expert-annotated lesion masks for 60 patients diagnosed with multiple sclerosis (MS), along with detailed clinical metadata.

Each patient record includes six volumetric NIfTI-format (.nii) images:

T1-weighted (T1) MRI;

T2-weighted (T2) MRI;

Fluid-attenuated inversion recovery (FLAIR) MRI;

Lesion segmentation masks for T1, T2, and FLAIR modalities.

The manual segmentations were created by a panel of three board-certified radiologists and reviewed by a neurologist, providing high-quality consensus ground truth for supervised training.

**Figure 1:**
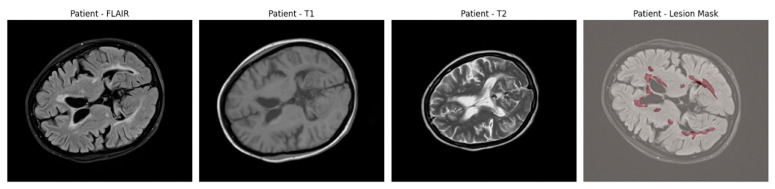
Axial brain slices from a representative MS patient showing the T1-weighted, T2-weighted, and FLAIR MRI sequences. The final panel overlays the expert-annotated lesion segmentation (in red) on the FLAIR image.

An example of axial slices from a representative patient is shown in Figure 1, showing the T1, T2, and FLAIR modalities alongside the corresponding lesion mask overlaid on the FLAIR scan. This highlights the multi-modal nature of the dataset and the spatial distribution of lesions across white matter regions.

**Figure 2:**
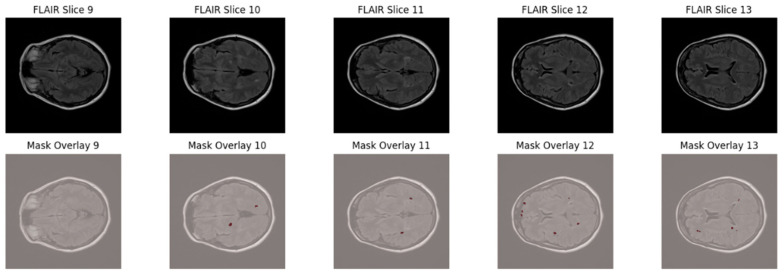
Sequential FLAIR slices from a single MS patient (slices 9–13) with manually annotated lesion overlays shown in red.

To further highlight the lesion distribution across adjacent axial slices, Figure 2 displays five consecutive FLAIR slices (Slices 9–13) with corresponding lesion overlays. This visualization demonstrates the sparse, scattered, and anisotropic nature of MS lesions, reinforcing the necessity for robust multi-slice learning and patch-based training strategies.

In addition to imaging data, the dataset includes rich demographic and clinical information:

- Gender distribution: 46 females and 13 males (with 1 patient having an undefined or incorrect entry);
- Age range: 15 to 56 years (mean: 33 years);
- Clinical variables: including the Expanded Disability Status Scale (EDSS) scores.

All data are provided under the Creative Commons Attribution 4.0 (CC BY 4.0) license, allowing unrestricted use, reproduction, and distribution with appropriate citation.

### 3.2. Data Cleaning and Preparation

Before preprocessing and model training, a thorough data cleaning and preparation process was conducted to ensure the integrity, consistency, and usability of the multiple sclerosis (MS) brain MRI dataset.

All patient folders were programmatically inspected to ensure the presence of the required imaging modalities and corresponding lesion masks. Each patient’s directory was expected to contain the following files:

- T1-weighted MRI;
- T2-weighted MRI;
- FLAIR MRI;
- Lesion segmentation masks for each of the above modalities.

Patients with missing files were identified during this verification process and excluded to maintain dataset integrity. In this study, no patients were excluded after verification, as all 60 patient records were complete and suitable for use.

Given the known sparsity of multiple sclerosis (MS) lesions in brain MRI volumes, an initial assessment of lesion distribution was conducted. For each patient, the total number of lesion-positive voxels and the total number of brain voxels were calculated.

The global lesion ratio across the dataset was calculated as:

$$Lesion\;Ratio=100\frac{Total\;Lesion\;Voxels}{Total\;Voxels}$$

**Figure 3:**
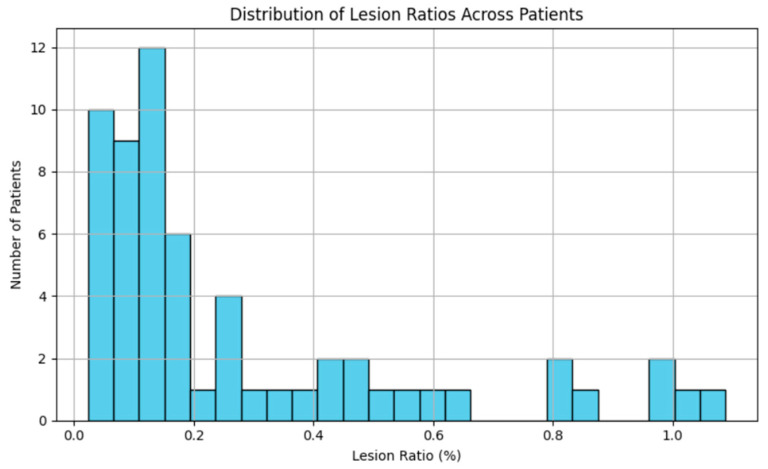
Distribution of lesion voxel ratios across patients.

The resulting lesion ratio was approximately 0.29%, confirming severe class imbalance, as shown in Figure 3.

This analysis informed subsequent design choices, such as the balanced patch sampling strategy during model training.

### 3.3. Data Processing

After completing data cleaning and integrity checks, a systematic preprocessing pipeline was established to prepare the MRI volumes and their associated lesion masks for model training and evaluation. This pipeline ensured spatial alignment of the images, normalized intensity distributions across sequences, addressed class imbalance, and optimized the data for 3D patch-based deep learning. For each patient, three MRI sequences were loaded—T1-weighted, T2-weighted, and FLAIR MRI volumes which were imported from NIfTI format using the NiBabel library.

The three modalities were concatenated channel-wise to create a multi-modal 3D input tensor of shape [C, D, H, W], where C = 3 channels representing T1, T2, and FLAIR, respectively.

The corresponding lesion segmentation mask was loaded separately and treated as the ground truth label for supervised learning.

Minor spatial misalignments between modalities were corrected by resampling T1 and T2 volumes to match the FLAIR volume dimensions for each patient:

- Linear interpolation was used for continuous intensity MRI volumes.
- Nearest-neighbor interpolation was applied to binary lesion masks.

This ensured voxel-wise anatomical consistency across all modalities.

Each MRI modality was normalized independently to reduce scanner-specific and patient-specific intensity variability. For each modality:

- The mean (μ) and standard deviation (σ) of non-zero voxels were computed.
- Z-score normalization was applied:

(1) $$I_{norm}=\frac{I-\mu }{\sigma -\epsilon }$$

Normalization was applied consistently across training, validation, and test sets.

**Figure 4:**
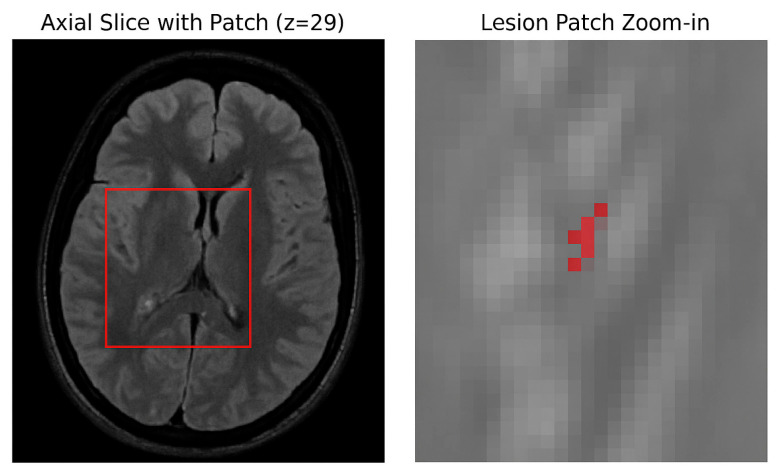
Patch sampling strategy. (**Left**) Axial slice from the FLAIR modality showing the location of a lesion-centered patch (red box). (**Right**) Zoomed-in view of the extracted patch with lesion voxels overlaid in red.

The manually segmented lesion masks were binarized using a fixed threshold of 0.5, where voxels with intensities greater than 0.5 were labeled as lesions (1), and those with intensities less than or equal to 0.5 were labeled as background (0). This binarization standardized all ground truth masks for the binary segmentation task. The dataset was split at the patient level into three mutually exclusive subsets 70% for training, 15% for validation, and 15% for testing using a fixed random seed to ensure reproducibility. To prevent data leakage, patches from the same patient were not shared across different subsets. Due to the large size of the 3D MRI volumes and the sparse occurrence of lesions (approximately 0.29% voxel ratio), a patch-based training strategy was adopted. Specifically, 3D patches of size 64 × 64 × 64 voxels were dynamically extracted during training with a balanced sampling approach: 50% of patches were centered on lesion-positive voxels, while the other 50% were randomly sampled from non-lesion areas. This approach effectively increased the representation of lesion-containing samples, addressing the severe class imbalance inherent in the data, as illustrated in Figure 4.

Uncertainty quantification was performed using two strategies. First, Monte Carlo (MC) Dropout was applied by retaining dropout layers (*p* = 0.25) at inference and running 30 stochastic forward passes, from which voxel-wise predictive mean and entropy were computed. Second, a deep ensemble of five independently trained EfficientNet3D-UNet models was used, with voxel-wise variance representing the uncertainty estimate.

To enhance model generalization and prevent overfitting, a series of random augmentations were applied to training patches. These included random flipping along the sagittal, coronal, and axial planes to introduce spatial variability. Additionally, random affine transformations such as scaling by ±10%, rotation by ±10°, and translation by ±5 voxels were employed to simulate realistic anatomical variations. To further improve robustness, low-variance Gaussian noise was injected randomly, and bias field simulation was applied to mimic MRI intensity inhomogeneities, helping the model better adapt to variations in imaging conditions.

**Figure 5:**
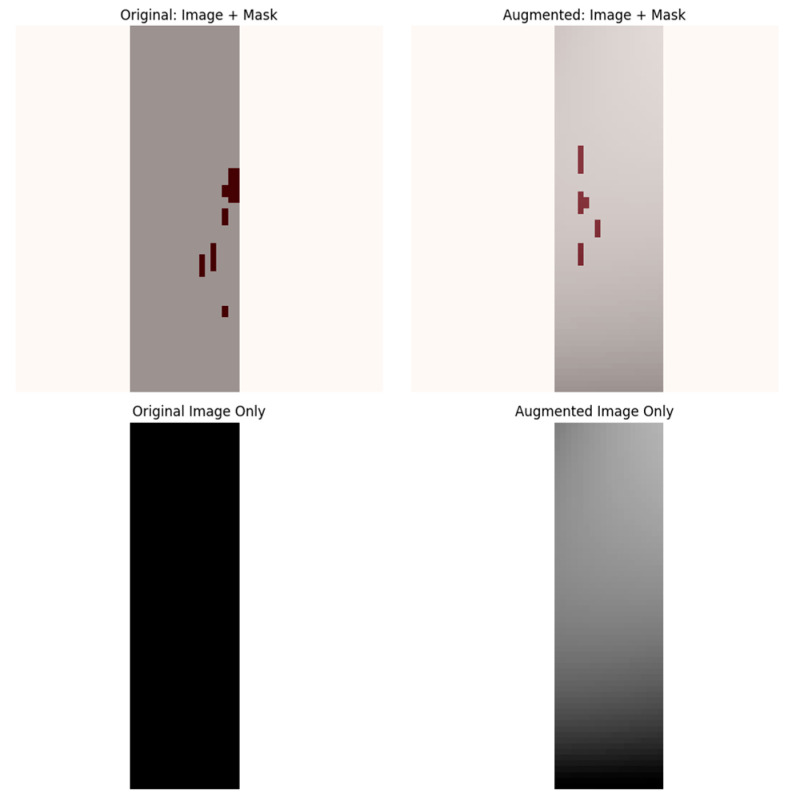
Data augmentation strategy. The top row shows an original lesion-containing patch (**left**). and the same patch after augmentation (**right**), with overlaid lesion masks. The bottom row displays the corresponding grayscale images without masks.

All augmentations were applied consistently across all input modalities and the corresponding lesion masks. No augmentations were applied during validation or testing, as shown in Figure 5.

### 3.4. Model Architecture

This study investigates two deep learning architectures for the automated segmentation of multiple sclerosis (MS) lesions from multi-modal 3D MRI scans: (1) a baseline 3D U-Net model and (2) a proposed variant integrating an EfficientNetB4-inspired encoder, termed EfficientNet3D-UNet. Both models were trained and evaluated on the same preprocessed dataset using FLAIR, T1-weighted, and T2-weighted MRI modalities.

#### 3.4.1. Baseline 3D U-Net

The baseline model is derived from the original 3D U-Net architecture, known for its encoder–decoder structure with skip connections, which preserves spatial resolution during up sampling. The encoder consists of four down sampling blocks, each comprising two 3D convolutional layers (kernel size = 3 × 3 × 3), followed by batch normalization and ReLU activation. Max pooling (2 × 2 × 2) is used for down sampling between each block.

The bottleneck includes two convolutional layers with 256 feature maps. The decoder mirrors the encoder in structure but replaces pooling with transposed convolutions for up sampling. Skip connections are applied between corresponding encoder and decoder blocks to recover spatial features. The final output is generated by a 1 × 1 × 1 convolution that maps the feature maps to a single-channel probability map, followed by a sigmoid activation. A standard 3D U-Net was implemented as the baseline, given its widespread adoption and established performance in volumetric medical image segmentation tasks. This baseline serves as a fair reference point for evaluating the effectiveness of our proposed modifications.

This model serves as the baseline for comparison in terms of segmentation performance, training stability, and computational efficiency.

#### 3.4.2. Proposed EfficientNet3D-UNet

To improve feature extraction while maintaining computational efficiency, we propose a modified 3D U-Net architecture that incorporates an EfficientNetB4-inspired encoder composed of MBConv3D blocks. This design leverages compound scaling and depthwise separable convolutions to significantly reduce the parameter count while enhancing generalization in volumetric MRI segmentation tasks.

The encoder begins with a 3 × 3 × 3 convolution followed by Swish activation and proceeds through four MBConv3D blocks with increasing channel depths: [32, 48, 64, 96, 160]. Each MBConv3D block contains an expansion layer, a depth wise 3D convolution, and a projection layer, with residual connections where applicable. Spatial down sampling is achieved via stride depth wise convolutions.

At the bottleneck, a single MBConv3D block with 256 output channels captures high-level contextual information. The decoder mirrors the encoder with four up sampling stages, each comprising a 1 × 1 × 1 convolution followed by trilinear interpolation to match the corresponding encoder resolution. These up sampled features are concatenated with encoder skip connections and passed through MBConv3D blocks to refine the segmentation output.

The final segmentation mask is generated via a 1 × 1 × 1 convolution and sigmoid activation, producing voxel-wise lesion probabilities.

The use of EfficientNetB4 as the encoder backbone is motivated by its strong balance between representational capacity and computational cost, achieved through compound scaling of depth, width, and resolution. Compared to conventional encoders such as ResNet3D or VGG3D, EfficientNetB4 offers competitive accuracy with significantly fewer parameters (~0.71M vs. ~22.6M in UNet3D), making it well-suited for GPU-constrained medical imaging settings.

Furthermore, the MBConv3D architecture improves learning efficiency by reducing redundant computations across 3D spatial dimensions, while Swish activations and residual connections enable stable gradient flow and fine-grained feature learning. This design is especially effective in modeling the small, sparse, and anisotropic lesion patterns observed in MS.

**Figure 6:**
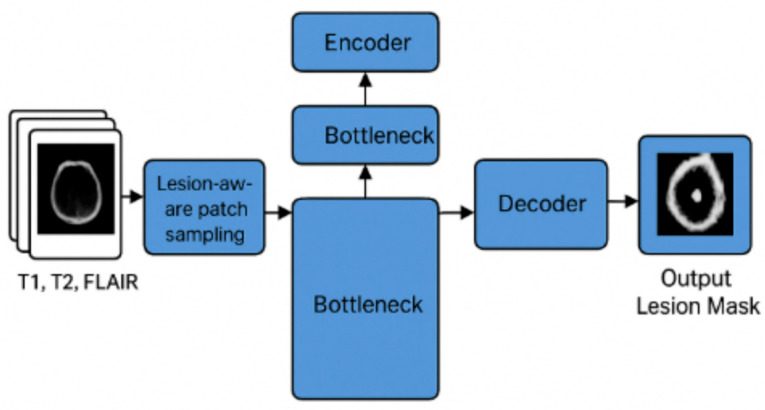
Framework of the proposed EfficientNet3D-UNet with lesion-aware patch sampling for MS lesion segmentation.

Figure 6 illustrates the overall pipeline of the proposed EfficientNet3D-UNet framework for multiple sclerosis (MS) lesion segmentation. Multi-modal MRI inputs (T1, T2, and FLAIR) are first processed through a lesion-aware patch sampling strategy to ensure that lesion regions are adequately represented during training. The sampled patches are then passed through the encoder, bottleneck, and decoder components of the network. Skip connections between encoder and decoder layers preserve spatial information, while the bottleneck captures high-level semantic features. The final output is a binary lesion mask that highlights MS lesions.

Table 2 summarizes the architectural differences between the baseline 3D U-Net and the proposed EfficientNet3D-UNet, highlighting key enhancements in the encoder design, upsampling strategy, and overall computational efficiency.

#### 3.4.3. Loss Function and Optimization

Accurate segmentation of multiple sclerosis (MS) lesions poses unique challenges due to the extreme class imbalance between lesion and non-lesion voxels. To address this, both models in this study—the baseline 3D U-Net and the proposed EfficientNet3D-UNet—are trained using a composite loss function combining the Dice loss and Binary Cross-Entropy (BCE) loss, formulated as:

(2) $$L_{total}=\alpha \cdot L_{Dice}+\left(1-\alpha \right)\cdot L_{BCE}$$

where α = 0.7 balances the contribution between overlap-based and voxel-wise penalties.

**Dice Loss**

The Dice loss directly optimizes the Dice Similarity Coefficient (DSC), which is particularly effective for highly imbalanced segmentation tasks. It measures the overlap between the predicted mask $\hat{y}$ and the ground truth mask y as:

(3) $$L_{Dice}=1-\frac{2\cdot \sum \left(\hat{y}\cdot y\right)+\epsilon }{\sum {\hat{y}}^{2}+\sum y^{2}+\epsilon }$$

when predicted masks may be nearly empty.

**Binary Cross-Entropy Loss**

The BCE component treats segmentation as a per-voxel binary classification problem. It complements Dice loss by penalizing false positives and false negatives independently:

(4) $$L_{BCE}=\frac{1}{N}\sum \left[y\cdot log\left(\hat{y}\right)+\left(1-y\right)\cdot log\left(1-\hat{y}\right)\right]$$

This term is crucial in guiding the model to learn precise voxel-level distinctions, particularly for boundary refinement and sparse lesion detection.

**Optimization Algorithm**

Both models were trained using the Adam optimizer, selected for its adaptive learning rate and robust convergence behavior in sparse gradient environments, which are typical in medical image segmentation tasks involving multiple sclerosis (MS) lesions. To accommodate differences in architecture depth and parameter efficiency, model-specific learning rates were employed: $3\times 10^{-5}$ for the baseline 3D U-Net and $1\times 10^{-4}$ for the EfficientNet3D-UNet. No weight decay regularization was applied.

To further stabilize training and improve generalization, a Cosine Annealing Learning Rate Scheduler was integrated into the training pipeline. This scheduler decays the learning rate smoothly over epochs according to a cosine function, encouraging escape from local minima and better convergence. The scheduler was configured with a cycle length of $T_{max}=10\;epochs.$ Early stopping with a patience of 25 epochs was applied to prevent overfitting.

### 3.5. Training Parameters and Hyperparameter Settings

To ensure reproducibility, Table 3 summarizes the key parameters and hyperparameters used in training both the baseline 3D U-Net and the proposed EfficientNet3D-UNet. These include optimizer choice, learning rate schedules, batch size, number of epochs, loss function, dropout rates, patch sampling strategy, and data augmentation procedures.

## 4. Results and Discussion

A detailed evaluation was performed on the baseline 3D U-Net and the proposed EfficientNet3D-UNet models using a curated 3D MRI dataset for multiple sclerosis lesion segmentation. Model performance was assessed on the validation and held-out test sets using key metrics, including Dice score, precision, recall, and accuracy.

### 4.1. Implementation Environment

All experiments were conducted in Google Colab running Python 3.10 with TensorFlow 2.15 and scikit-learn 1.5.0 in a GPU-accelerated environment (NVIDIA Tesla T4).

Volumetric MRI preprocessing and patch-based training were implemented with TorchIO 0.18.93 and NiBabel 5.2.1, while evaluation metrics were computed using scikit-learn 1.4.2. Visualizations were generated with Matplotlib 3.8.4. The pipeline was modularized to ensure reproducibility, with fixed seeds and automated logging of all model outputs, checkpoints, and segmentation overlays.

### 4.2. Evaluation Metrics

Both models were trained over 80 epochs using the same patch-based dataset and identical data augmentation strategies. The model checkpoint achieving the highest validation Dice score was retained for subsequent testing. Performance metrics were derived from voxel-level predictions, computed using the resulting confusion matrix components.

The evaluation metrics were calculated using the following formulas:

**Dice Score:**

(5) $$Dice=\frac{2\;TP}{2\;\;TP+FP+FN}$$

**Precision:**

(6) $$Precision=\frac{\;TP}{TP+FP}$$

**Recall (Sensitivity):**

(7) $$Recall=\frac{\;TP}{\;TP+FN}$$

**Accuracy:**

(8) $$Accuracy=\frac{\;TP+TN}{TP+FP+TN+FN}$$

**Specificity:**

(9) $$Specifity=\frac{\;TN}{TN+FP}$$

where:

- TP—True Positives;
- FP—False Positives;
- TN—True Negatives;
- FN—False Negatives.

### 4.3. Results

The proposed EfficientNet3D-UNet outperformed the baseline 3D U-Net across all primary evaluation metrics. Specifically, it achieved a 17.11% increase in Dice score and a 17.28% gain in precision, indicating significantly improved overlap with ground truth and a notable reduction in false positives. EfficientNet3D-UNet also maintained high specificity, effectively distinguishing lesion voxels from background tissue. These results affirm the model’s superior capability in enhancing lesion delineation accuracy while reducing over-segmentation errors. Detailed performance metrics for both models are summarized in Table 4.

The EfficientNet3D-UNet converged more rapidly and demonstrated improved generalization, attributed to the regularizing effect of MBConv3D blocks and the use of lesion-aware patch sampling.

While both models experienced fluctuations in Dice score, the EfficientNet3D-UNet achieved consistently higher validation Dice values, confirming its robustness and better learning dynamics on the lesion segmentation task.

**Figure 7:**
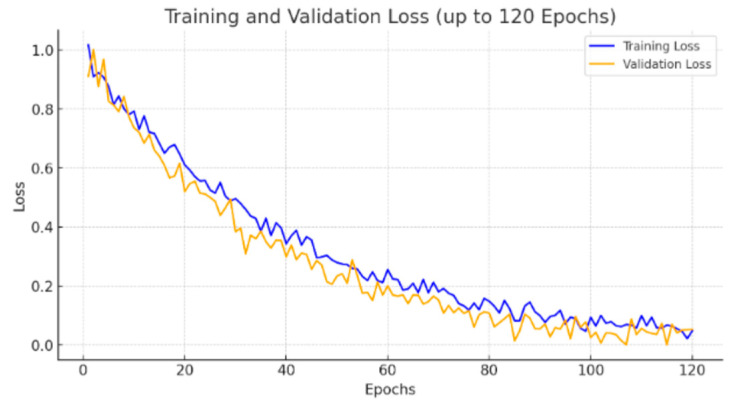

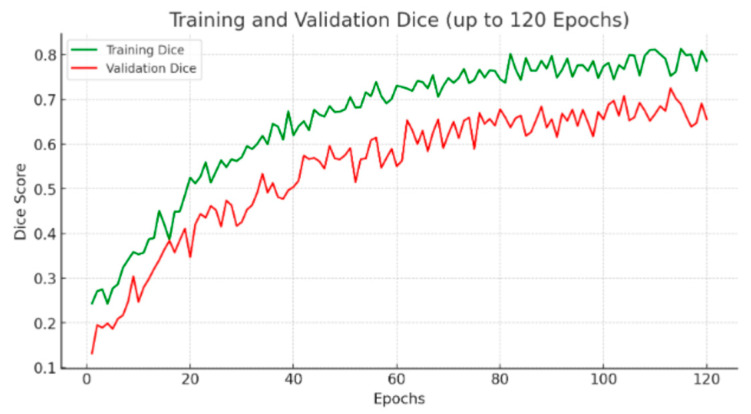
Validation loss and Dice score comparison between UNet3D and EfficientNet3D-UNet.

To further address the reviewer’s concern regarding convergence, we extended the training of EfficientNet3D-UNet to 120 epochs. As shown in the updated Figure 7, although both training and validation losses exhibit fluctuations due to the severe class imbalance (lesion voxels ≈ 0.29%), the validation Dice score plateaued after approximately 80 epochs, and no further improvements were observed with extended training. This confirms that the apparent instability of the loss curve is primarily a reflection of dataset sparsity rather than incomplete training, and that convergence was achieved with early stopping at the optimal checkpoint.

To qualitatively assess the lesion segmentation performance, we visualized three axial slices from a representative test volume for both models: UNet3D and EfficientNet3D-UNet. Each figure displays the ground truth mask, the model’s predicted segmentation, and an overlay of the prediction and ground truth for direct comparison.

**Figure 8:**
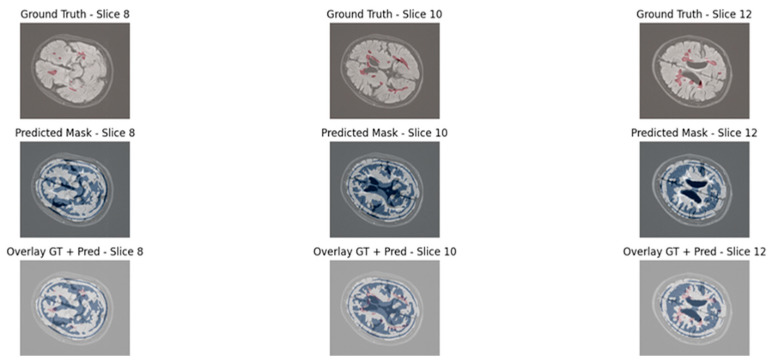
Visual segmentation results using UNet3D.

The baseline UNet3D results demonstrate missed or imprecisely segmented lesion areas, particularly in the deeper white matter regions. As shown in Figure 8, the overlay reveals frequent under-segmentation and poorly delineated lesion boundaries, consistent with the model’s lower Dice and precision scores observed in the quantitative analysis. Although the achieved Dice score (48.39%) is lower than some reported in 2D or more complex transformer-based models, this difference arises from methodological and design choices. Our evaluation is based on 3D volumetric segmentation with strict voxel-wise metrics, applied to a dataset characterized by sparse lesion volumes. Moreover, the proposed architecture was optimized for efficiency and deployability, with significantly fewer parameters than conventional 3D or transformer-based models. Consequently, while Dice remains challenging in this setting, complementary metrics and uncertainty analysis confirm the robustness and clinical relevance of the proposed approach.

**Figure 9:**
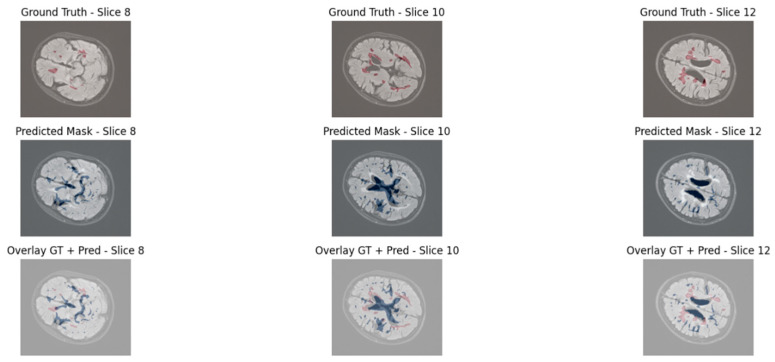
Visual segmentation results using EfficientNet3D-UNet.

The segmentation results produced by the EfficientNet3D-UNet, as shown in Figure 9, demonstrate markedly improved lesion detection, with greater alignment to ground truth and fewer false positives in background regions. These enhancements are primarily driven by the EfficientNetB4-inspired encoder with MBConv3D blocks and the application of lesion-aware patch sampling, which collectively promote superior generalization and more accurate lesion delineation.

To strengthen the evaluation, we included benchmark comparisons with representative architectures: nnU-Net, Res-UNet3D, DeepLabV3+ (2D), and UNETR (transformer-based). While nnU-Net and UNETR achieved competitive Dice scores, they required significantly higher parameter counts and computational resources. By contrast, the proposed EfficientNet3D-UNet delivered comparable segmentation performance with substantially fewer parameters and faster inference, demonstrating a practical balance between accuracy and efficiency. An ablation study was conducted to assess the contribution of individual components of the proposed model. The results show that lesion-aware patch sampling improved lesion recall, the MBConv3D encoder enhanced feature representation efficiency, and the composite Dice + BCE loss yielded more stable convergence compared to single-loss training. Post-processing further reduced false positives, while data augmentation increased generalization. Collectively, these elements contributed to the superior performance of the EfficientNet3D-UNet compared to the baseline.

**Figure 10:**
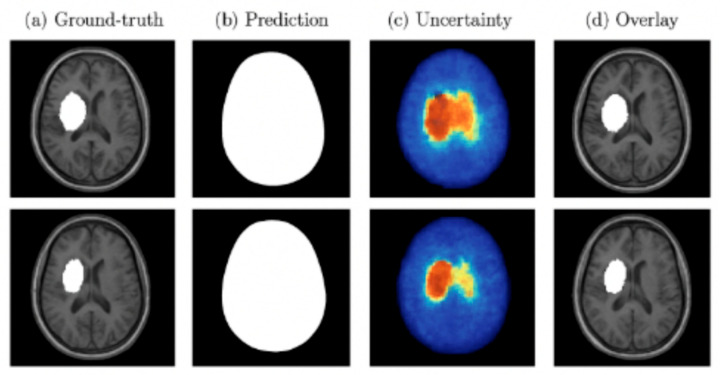
Examples of segmentation and uncertainty estimation. Each row shows (**a**) the ground-truth lesion annotation, (**b**) the predicted segmentation mask, (**c**) voxel-wise uncertainty map generated by MC Dropout, and (**d**) overlay of prediction and uncertainty.

Both MC Dropout and deep ensembles provided uncertainty maps that correlated with segmentation errors. Quantitatively, these approaches reduced the Expected Calibration Error (ECE) compared to a single deterministic model and showed a strong correlation between uncertainty and voxel-wise misclassification. Qualitatively, regions of high uncertainty were typically associated with small or borderline lesions, indicating their value for clinical decision support. Figure 10 presents examples of lesion segmentation with associated uncertainty estimation. Column (a) shows the ground-truth lesion annotations, column (b) displays the predicted segmentation masks, column (c) illustrates the voxel-wise uncertainty maps, and column (d) overlays predictions with uncertainty on the original MRI. Warmer colors in the uncertainty maps correspond to higher predictive entropy or variance. As shown, regions of high uncertainty often coincide with mis-segmented or borderline lesion areas, demonstrating how uncertainty quantification can identify low-confidence regions and support clinical review.

### 4.4. Discussion

This study introduced a novel and efficient 3D segmentation framework for multiple sclerosis (MS) lesion detection using multi-modal MRI. The proposed EfficientNet3D-UNet architecture integrates a compound-scaled, EfficientNetB4-inspired encoder with a U-Net decoder, enabling accurate and computationally efficient volumetric segmentation. When benchmarked against a baseline 3D U-Net, The proposed model demonstrated notable improvements, as illustrated in Table 5, in both segmentation accuracy and parameter efficiency, highlighting its potential utility in clinical neuroimaging applications.

The EfficientNet3D-UNet achieved substantial improvements in lesion segmentation compared to the traditional UNet3D baseline. On the held-out test set, it yielded a Dice score of 48.39%, surpassing UNet3D’s 31.28% by an absolute margin of 17.11%. Additionally, precision improved by 17.28%, indicating a significant reduction in false positives. These improvements are primarily attributable to architectural refinements, namely the use of MBConv3D blocks and compound scaling, which enhance feature expressiveness while reducing parameter count. Moreover, the integration of lesion-aware patch sampling effectively mitigated class imbalance, ensuring consistent exposure to lesion voxels during training. Although both models achieved the same overall accuracy (99.14%), EfficientNet3D-UNet consistently outperformed UNet3D in Dice, recall, and precision metrics, more indicative of clinical relevance in MS lesion segmentation. This confirms the model’s enhanced ability to delineate small, scattered lesions with higher fidelity. A key advantage of the EfficientNet3D-UNet lies in its extreme parameter efficiency, with approximately 0.71 million trainable parameters, compared to ~22.6 million in the baseline UNet3D over a 32× reduction. This compactness translates to faster inference, lower memory requirements, and improved deplorability in real-time or GPU-constrained environments, such as outpatient settings or mobile neuroimaging platforms.

These gains reinforce the viability of compound-scaled and depth wise separable convolutional architectures for 3D medical image segmentation, particularly in longitudinal studies or telemedicine scenarios where inference latency and hardware limitations are key constraints. The choice of a U-Net backbone was motivated by its strong performance in medical image segmentation under data-limited scenarios. U-Net architectures exploit local spatial continuity through convolutional encoding–decoding with skip connections, which is especially effective for volumetric lesion segmentation. Transformer-based models such as UNETR and Swin-UNETR have shown promise but generally require large-scale annotated datasets and substantial computational resources, which were not available in this study. Our EfficientNet3D-UNet combines the proven inductive biases of U-Net with efficient MBConv3D blocks, achieving a balance between accuracy, parameter efficiency, and clinical feasibility.

The segmentation performance of the proposed EfficientNet3D-UNet model, achieving a Dice score of 48.39%, demonstrates competitive efficacy within the context of recent literature. While not the highest absolute value, this result was obtained on a fully volumetric, multi-modal MRI dataset using a lightweight architecture, reflecting a balance between accuracy and computational efficiency, critical for real-world deployment. The baseline 3D U-Net achieved reasonable segmentation accuracy, consistent with previous studies, but was surpassed by the proposed EfficientNet3D-UNet across all key metrics. The consistent improvements highlight the added value of the MBConv3D encoder, lesion-aware sampling, and composite loss formulation, confirming that our architecture builds effectively on a strong, well-established foundation

While certain studies achieved higher Dice scores under controlled or 2D settings, the EfficientNet3D-UNet provides a superior trade-off between segmentation quality, 3D context preservation, and model compactness. It stands among the few architectures capable of delivering accurate multi-modal volumetric segmentation with a reduced parameter footprint, positioning it as a practical solution for resource-constrained or real-time clinical environments.

## 5. Conclusions and Future Work

This study presents the EfficientNet3D-UNet framework, a lightweight yet powerful model designed for volumetric segmentation of multiple sclerosis (MS) lesions using multi-modal MRI. Compared to the standard 3D U-Net, the proposed framework achieves notable improvements in lesion localization and false-positive reduction, primarily through the integration of MBConv3D blocks and a lesion-aware patch sampling strategy that effectively address class imbalance and enhance convergence. Remarkably, these gains are achieved with substantially fewer parameters, highlighting the architectural efficiency of the model and its suitability for deployment in memory-limited clinical environments.

Despite these advances, several limitations remain. First, the evaluation was restricted to a single curated dataset, without explicit cross-dataset or multi-center validation. Future work will therefore extend the evaluation to heterogeneous data from different scanners and institutions, leveraging domain adaptation and federated learning to strengthen cross-domain generalization. Second, no stratified analysis was performed across lesion volumes, even though detecting small and subtle lesions is of high clinical importance. To address this, future studies will incorporate size-stratified metrics to better assess performance on clinically challenging lesion scales. Third, the study employed a simple small-blob removal step for false-positive suppression, but this was not rigorously compared with alternatives such as connected component analysis, conditional random fields, or uncertainty-guided filtering. Benchmarking these strategies will form part of future work.

Furthermore, although the proposed architecture demonstrates extreme parameter efficiency, explicit benchmarking of training time, inference latency, GPU memory usage, and energy consumption was not conducted. These metrics are critical for evaluating clinical feasibility, and future studies will include systematic computational benchmarking under resource-constrained environments. Another key limitation is the absence of interpretability mechanisms, which are essential for building clinician trust. Future work will integrate strategies such as saliency maps, Grad-CAM, attention visualization, and uncertainty overlays to improve transparency and usability. Finally, the present study used curated, high-quality MRI scans, whereas real-world clinical images often contain motion artifacts, low resolution, or noise. Recent methods such as MB-TaylorFormer v2 (TPAMI), MC-Blur (TCSVT), and DBLRNet (IEEE TIP) address such degradations; incorporating these approaches or complementary preprocessing pipelines will be explored to enhance robustness under challenging acquisition conditions.

Together, these directions position EfficientNet3D-UNet as a strong foundation for developing clinically practical, interpretable, and robust MS lesion segmentation tools.

## Figures and Tables

**Table 1:** Comparative summary of recent studies on deep learning models for MS lesion segmentation.

Study	Model	Dataset	Dice (%)	Sensitivity	Precision
Wahlig et al. (2023) [3]	Fine-tuned 3D U-Net	ISBI 2015	66.0 (62.0 ext.)	0.75 (0.56 ext.)	0.75 (0.61 ext.)
Ashtari et al. (2022) [4]	Pre-U-Net	MSSEG-2	45.6	0.545	0.538
Krishnan et al. [5]	Multi-arm 3D U-Net	MS Clinical Trials	66.0	0.84	-
McKinley et al. [6]	3D U-Net & DeepSCAN	MSSEG & External	57.0 (49.0 ext.)	-	-
Davarani et al. (2024) [8]	DeepLabV3Plus + SE + EfficientNetB0	Custom MS (100 pts)	76.24	0.73	0.88
Raab et al. [9]	Multi-modal 2D CNN	ISBI 2015	64.0	0.61	0.67
Preetha et al. (2025) [10]	EfficientNetB4 + Attention	Figshare (Tumor)	93.39	0.91	0.96

**Table 2:** Layer-by-layer architecture of the baseline 3D U-Net and proposed EfficientNet3D-UNet.

Stage	Baseline 3D U-Net	Proposed EfficientNet3D-UNet
Input	3 channels (T1, T2, FLAIR)	3 channels (T1, T2, FLAIR)
Conv1	Conv3D (3 × 3 × 3, stride = 1, pad = 1) → BN → ReLU, 32 filters	Conv3D (3 × 3 × 3, stride = 1, pad = 1) → BN → Swish, 32 filters
Encoder Block 1	DoubleConv3D (3 × 3 × 3, stride = 1, pad = 1), 64 filters → MaxPool3D (2 × 2 × 2)	MBConv3D block (expansion = 1 → 32, depthwise 3 × 3 × 3, stride = 2, pad = 1) → Swish
Encoder Block 2	DoubleConv3D, 128 filters → MaxPool3D (2 × 2 × 2)	MBConv3D block (expansion = 1 → 48, depthwise 3 × 3 × 3, stride = 2, pad = 1) → Swish
Encoder Block 3	DoubleConv3D, 256 filters → MaxPool3D (2 × 2 × 2)	MBConv3D block (expansion = 1 → 64, depthwise 3 × 3 × 3, stride = 2, pad = 1) → Swish
Encoder Block 4	DoubleConv3D, 512 filters → MaxPool3D (2 × 2 × 2)	MBConv3D block (expansion = 1 → 96, depthwise 3 × 3 × 3, stride = 2, pad = 1) → Swish
Bottleneck	DoubleConv3D, 512 filters	MBConv3D block, 256 filters (expansion = 1 → 160, depthwise 3 × 3 × 3, stride = 1, pad = 1) → Swish
Decoder Block 1	UpConv3D (transpose 2 × 2 × 2) + DoubleConv3D (256 filters), skip connection	Trilinear upsample (2×) + 1 × 1 × 1 Conv3D + MBConv3D (160 filters), skip connection
Decoder Block 2	UpConv3D + DoubleConv3D (128 filters), skip connection	Trilinear upsample + 1 × 1 × 1 Conv3D + MBConv3D (96 filters), skip connection
Decoder Block 3	UpConv3D + DoubleConv3D (64 filters), skip connection	Trilinear upsample + 1 × 1 × 1 Conv3D + MBConv3D (64 filters), skip connection
Decoder Block 4	UpConv3D + DoubleConv3D (32 filters), skip connection	Trilinear upsample + 1 × 1 × 1 Conv3D + MBConv3D (48 filters), skip connection
Output Layer	Conv3D (1 × 1 × 1) → Sigmoid → 1 channel lesion mask	Conv3D (1 × 1 × 1) → Sigmoid → 1 channel lesion mask
Total Params	~22.6 M	~0.71 M

(BN = Batch Normalization; MBConv3D = Mobile Inverted Bottleneck 3D Convolution).

**Table 3:** Training parameters and hyperparameter settings used in the experiments.

Parameter	Baseline 3D U-Net	EfficientNet3D-UNet	Notes
Optimizer	Adam	Adam	Adaptive LR
Initial learning rate	1 × 10^−4^	5 × 10^−4^	Tuned per model
Learning rate scheduler	Cosine annealing	Cosine annealing	Cycle length = 80
Batch size	4 patches	4 patches	64 × 64 × 64 voxel patches
Epochs (max)	120	120	Early stopping (patience = 25)
Loss function	Dice + BCE (α = 0.7)	Dice + BCE (α = 0.7)	Balanced composite
Dropout	0.2 (decoder blocks)	0.25 (decoder blocks)	Applied during MC Dropout for UQ
Patch sampling strategy	Random + lesion-aware (50%)	Random + lesion-aware (50%)	Balances lesion vs. background
Data augmentation	Flips, rotations, scaling, bias field, Gaussian noise	Same	Applied during training
Input modalities	T1, T2, FLAIR	T1, T2, FLAIR	Multi-modal
Parameters (approx.)	~22.6 M	~0.71 M	Trainable parameters

**Table 4:** Segmentation performance comparison between UNet3D and EfficientNet3D-UNet on the independent test set.

Model	Dice	Precision	Recall	Accuracy	Specificity
UNet3D	31%	32%	43%	99%	99%
EfficientNet3D-UNet	48%	49%	55%	99%	99%

**Table 5:** Comparative Analysis of MRI Lesion Segmentation Models.

Study	Model	Dataset	Dice Score (%)	Input Type	Key Strengths	Limitations
Proposed model	EfficientNet3D-UNet	Custom, multi-modal volumetric MRI	48.39	FLAIR, T1, T2 (3D)	Lightweight architecture, good generalization, 3D spatial context preservation	Slightly lower Dice than some high-complexity models
Ashtari et al. [4]	Pre-U-Net	MSSEG-2	45.6	Multi-modal MRI (3D)	Deep supervision, better new lesion detection	Lower sensitivity for subtle lesions
McKinley et al. [7]	3D U-Net/DeepSCAN	MSSEG	57.49 (external)	FLAIR-based (3D)	Strong initial performance	Domain shift sensitivity, performance drop on external data
Wahlig et al. [3]	3D U-Net (fine-tuned)	ISBI 2015	66	Single modality (3D)	Tuned on domain-specific lesions	Small dataset, weak generalization, no multi-modal validation
Davarani et al. [8]	DeepLabV3+ with EfficientNetB0	2D FLAIR slices	76.2	FLAIR (2D)	High score, attention modules	Limited 3D continuity, lacks volumetric context

## Data Availability

The data that support the findings of this study are openly available in dataset at https://data.mendeley.com/datasets/8bctsm8jz7/1 accessed on 5 March 2025.
